# Nernstian Li^+^ intercalation into few-layer graphene and its use for the determination of K^+^ co-intercalation processes†

**DOI:** 10.1039/d0sc03226c

**Published:** 2020-10-08

**Authors:** Jingshu Hui, A. Nijamudheen, Dipobrato Sarbapalli, Chang Xia, Zihan Qu, Jose L. Mendoza-Cortes, Joaquín Rodríguez-López

**Affiliations:** Department of Chemistry, University of Illinois at Urbana–Champaign 600 South Mathews Avenue Urbana Illinois 61801 USA joaquinr@illinois.edu +1-217-300-7354; Department of Chemical & Biomedical Engineering, Florida A&M – Florida State University, Joint College of Engineering 2525 Pottsdamer Street Tallahassee Florida 32310 USA; Department of Materials Science and Engineering, University of Illinois at Urbana–Champaign 1304 West Green Street Urbana Illinois 61801 USA

## Abstract

Alkali ion intercalation is fundamental to battery technologies for a wide spectrum of potential applications that permeate our modern lifestyle, including portable electronics, electric vehicles, and the electric grid. In spite of its importance, the Nernstian nature of the charge transfer process describing lithiation of carbon has not been described previously. Here we use the ultrathin few-layer graphene (FLG) with micron-sized grains as a powerful platform for exploring intercalation and co-intercalation mechanisms of alkali ions with high versatility. Using voltammetric and chronoamperometric methods and bolstered by density functional theory (DFT) calculations, we show the kinetically facile co-intercalation of Li^+^ and K^+^ within an ultrathin FLG electrode. While changes in the solution concentration of Li^+^ lead to a displacement of the staging voltammetric signature with characteristic slopes *ca.* 54–58 mV per decade, modification of the K^+^/Li^+^ ratio in the electrolyte leads to distinct shifts in the voltammetric peaks for (de)intercalation, with a changing slope as low as *ca.* 30 mV per decade. Bulk ion diffusion coefficients in the carbon host, as measured using the potentiometric intermittent titration technique (PITT) were similarly sensitive to solution composition. DFT results showed that co-intercalation of Li^+^ and K^+^ within the same layer in FLG can form thermodynamically favorable systems. Calculated binding energies for co-intercalation systems increased with respect to the area of Li^+^-only domains and decreased with respect to the concentration of –K–Li– phases. While previous studies of co-intercalation on a graphitic anode typically focus on co-intercalation of solvents and one particular alkali ion, this is to the best of our knowledge the first study elucidating the intercalation behavior of two monovalent alkali ions. This study establishes ultrathin graphitic electrodes as an enabling electroanalytical platform to uncover thermodynamic and kinetic processes of ion intercalation with high versatility.

## Abstract 2

Nernstian signatures and swift voltammetry at graphene electrodes help elucidate alkali ion (co-)intercalation.

## Graphical abstract

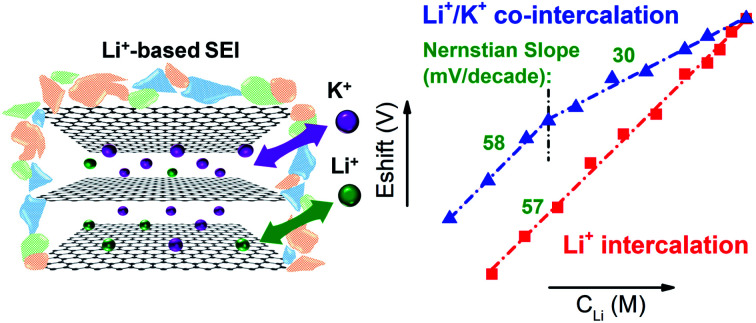

## Introduction

Alkali ion batteries (AIB) based on Li^+^, and emerging technologies based on K^+^, are important high-performance rechargeable energy storage devices.^1–3^ Considerable recent efforts have been devoted to the fabrication of new electrode materials,^4^ discovery of energy storage mechanisms,^5^ electrochemical reactions at electrodes and interphases,^6,7^ and towards improving the cell capacity, stability, and cyclability.^8^ Upon AIB cycling, alkali ions are reversibly stored within both anode and cathode in the battery. Therefore, microscopic understanding of the alkali ion intercalation processes under different external environments plays a vital role in deciphering the key aspects for future energy storage devices with improved performance.

Previous studies of alkali ion intercalation processes have unveiled the intercalation thermodynamics *via* slow scan rate cyclic voltammetry (CV)^9^ (*e.g*. at scan rates ≪ 1 mV s^−1^) and galvanostatic charge–discharge^7,10^ techniques. It is well-known that the Li^+^ intercalation process in graphitic materials follows a staging-type mechanism, where Li^+^ ions are progressively inserted within graphene planes to form multiple highly ordered layer structures, such as Stage 4 (LiC_36_), Stage 3 (LiC_27_), Stage 2 (LiC_12_), and Stage 1 (LiC_6_), respectively.^11,12^ Numerous research works focus on using various electrochemical, spectroscopic and scattering techniques to determine Li^+^ intercalation mechanisms as a bulk.^13–15^ Similarly to Li^+^, staging-type mechanisms have been described for K^+^ or Na^+^ as well,^10,16,17^ yet these systems still demonstrate limited device properties when compared to the well-established Li^+^ systems.^18^ Alkali ion co-intercalation with solvents such as diglymes is a commonly used strategy for activating Na^+^ and K^+^ intercalation,^19–21^ but consequently leads to exfoliation of the anode material and limits the number of sites for active ion storage.^22^ Li^+^ and K^+^ have been used as synergetic co-intercalation components to improve the sluggish Mg^2+^ intercalation kinetics on Li_4_Ti_5_O_12_,^23^ VS_4_ cathodes,^24^ and Ti_3_C_2_,^25^ V_2_C^26^ MXene anodes. However, to the best of our knowledge, the electrochemical co-intercalation of two alkali ions, and its corresponding voltammetric study, has not been demonstrated for graphitic carbon anodes.

Charge transfer in electrochemical systems is fundamentally explained by the Nernst equation and expressions deriving from it, as shown for the hypothetical process in eqn (1)*via*(2):

1 aA + bB + *n*e ⇌ cC

2
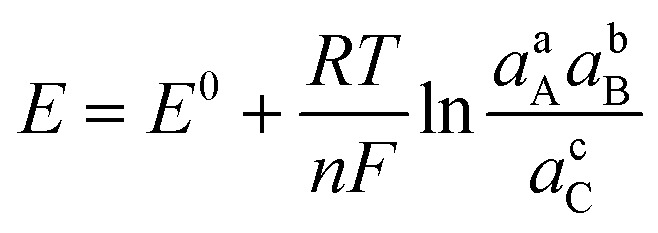
which relates the potential of a redox process (*E*) to a standard reduction potential (*E*^0^) and the logarithm of the reaction quotient expressed by the activities (*a*) of the involved species raised to their stoichiometric coefficients through the slope described by *T* the temperature, *R* the universal gas constant, *F* Faraday's constant and *n*, the number of electrons exchanged during reaction. The analysis of the Nernstian slope in eqn (2) displayed by voltammetric features as a function of species activity enables the experimenter to unravel mechanistic details of electrochemical reactions, including charge stoichiometry and ion-coupled electron transfer mechanisms. For instance, analysis of the response of solution and surface voltammetric peaks occurring as a function of pH, *i.e.* in systems depending on the activity of protons, is commonplace in electrocatalysis and molecular electrochemistry.^27,28^

Surprisingly, we could not find studies focused on experimentally determining that the Li^+^ intercalation charge transfer process responds in a Nernstian fashion. In fact, on the basis of open circuit voltage measurements in Li-ion batteries, discrepancies between the staging mechanism and the Nernstian description have been found for bulk graphite.^29,30^ Furthermore, knowledge gaps exist regarding the evaluation of charge transfer kinetics for ion intercalation at a microscopic scale; in part because this requires challenging electrode charge and discharge rates that are uncharacteristic in the battery literature. Our group recently reported on the mechanistic study of Li^+^ intercalation in electrodes made with few-layer graphene (FLG),^31^ as well as the preconditioned solid-electrolyte interphase (SEI) layer for facile K^+^ intercalation into this ultrathin graphitic carbon material.^16^ Mechanistic analysis using FLG electrodes is advantageous because there is no need to use extraneous materials such as binders. The geometry of these electrodes circumvents mass transfer limitations and enables voltammetric inspection at scan rates up to few V s^−1^. These rates are equivalent to 100–1000C (1C is fully discharge in 1 h), thus making the experimentation less cumbersome and enabling the direct exploration of kinetic limitations.^16^

Here, we turn to FLG electrodes as model interfaces to explore the Nernstian-type relationship of Li^+^ intercalation on a graphitic material. With this knowledge in hand, we show the signatures for Li^+^ and K^+^ co-intercalation, and explore for the first time the dependencies of the voltammetric staging behavior^9,32^ on the solution composition. Using CV analysis rooted in our Nernstian findings, we show that co-intercalation displays a single group of waves with two distinct behaviors for Li^+^-rich and K^+^-rich regions. We further determined their apparent alkali ion diffusion coefficients determined *via* potentiostatic intermittent titration technique (PITT),^33^ which were a function of Li^+^ content. We further studied the Li^+^ and K^+^ co-intercalation mechanism using periodic density functional theory (DFT) calculations. Through this approach, we were able to investigate the geometric changes, electronic structure tuning, and thermodynamic properties with respect to the Li^+^/K^+^ co-intercalation ratio. Combining theoretical calculations with experimental results, we propose a Li^+^/K^+^ ratio-dependent staging mechanism and calculated the apparent diffusion coefficient for the Li^+^ and K^+^ co-intercalation process. The present study shows a multi-faceted approach for identifying and predicting the thermodynamics and kinetics of alkali ion co-intercalation properties for advanced alkali ion-based energy storage. We believe the methodology described here can be extended to other systems where ion co-intercalation is suspected in order to understand mechanistic aspects of ion co-intercalation using Nernstian concepts.

## Experimental section

### Materials and supplies

All chemicals were purchased as A.C.S. reagent grade or better and used as received without further purification. Ethylene carbonate (EC, anhydrous, 99%), propylene carbonate (PC, anhydrous, 99.7%), lithium tetrafluoroborate (LiBF_4_, 99.99%, trace metals basis), potassium hexafluorophosphate (KPF_6_, 99.5%, trace metals basis), lithium hexafluorophosphate (LiPF_6_, 99.99%, trace metals basis) were obtained from Sigma-Aldrich. 3 inch Si wafer with 300 nm wet thermal oxide (Si/SiO_2_ wafer) was purchased from University Wafer. Few layer graphene (FLG) samples were fabricated *via* atmosphere pressure chemical vapor deposition (CVD) method with previously reported recipes.^16^

### Sample characterization

FLG samples were characterized through several techniques including scanning electron microscopy (SEM, Hitachi S-4800), Raman spectroscopy (Nanophoton Laser Raman Microscope RAMAN-11), and optical transmittance microscopy (Leica SP8 UV/Visible Laser Confocal Microscope). The optical transmittance image was obtained using a 561 nm laser line with constant intensity. Transmittance intensity of a blank glass cover slide was collected as reference (100% transmittance). The intensity at each pixel was then converted to percent transmittance, which is interpreted as a graphene layer number distribution map, by making use of a 2.3% decrease in optical transmittance per layer.^34^

### Electrochemical methods

Electrochemical measurements were performed in an Ar-filled drybox with oxygen and moisture levels less than 0.1 ppm using a CHI 760 potentiostat. The 1 : 1 (v/v) PC and EC mixture was used as the solvent in all tests, which is referred for simplicity as PC–EC in the main text. Three-electrode system was used in all tests, with a FLG working electrode (4.9 mm^2^), a Pt wire counter electrode (CE), and a Li strip or a Ag/Ag^+^ (saturated AgNO_3_ in PC–EC) reference electrode (RE). Potentials referenced against a Ag/Ag^+^ RE (3.725 V *vs.* 0.1 M Li/Li^+^) are reported *vs.* 0.1 M Li^+^/Li for clarity.^16^

#### Alkali ion (co-)intercalation characterization

The Li^+^ and K^+^ intercalation and co-intercalation behavior were characterized using cyclic voltammetry (CV). The Ag/Ag^+^ reference was used in all tests to obtain a stable reference potential, *i.e.* independent of Li^+^ or K^+^ concentration. Pristine FLG samples were first conditioned according to a previously reported procedure in 0.1 M LiBF_4_ PC–EC to form a robust Li^+^-based SEI layer yielding reproducible voltammetric signatures.^16^ The FLG samples were then characterized in 1 mL 0.1 M LiBF_4_ PC–EC solution and/or 0.1 M KPF_6_ PC–EC solution at various scan rates to examine the Li^+^ and K^+^ (co-)intercalation behavior. Samples tested in multiple solutions were thoroughly rinsed six times with PC in between experiments to fully remove the previous electrolyte. The Li^+^ Nernstian-relationship was tested by spiking 0.1 M TBAPF_6_ into 1 mL of 0.1 M LiPF_6_ PC–EC solution to obtain various Li^+^ concentration without affecting the solution conductivity. Similarly, the co-intercalation tests of both Li^+^ and K^+^ were obtained by spiking 0.1 M LiPF_6_ into 1 mL of 0.1 M KPF_6_ PC–EC solution to obtain various Li^+^/K^+^ ratio.

#### Diffusion coefficient determination

The diffusion coefficients of Li^+^ and K^+^ in the FLG electrodes, *D*_Li^+^_ and *D*_K^+^_ as a function of composition were obtained *via* PITT, which is a chronoamperometric method for evaluating ion diffusivity in the host at given potential increments.^35^ The typical PITT potential regions were guided by CV results of Li^+^/K^+^ co-intercalation with 5 mV potential intervals. An example of stage notations, representative regions and the resulting PITT chronoamperograms are found in Fig. S1 and S2.† The duration of each titration step was 150 s, 240 s, and 300 s for the background, FLG to Stage 3, and Stage 3 to Stage 1 regions, respectively (Fig. S1b†). The diffusion coefficient at each potential was estimated using eqn (3):^36^3
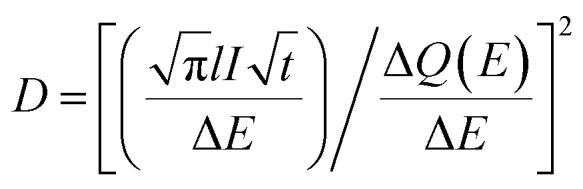
where *D* is the ion diffusion coefficient in the host, *l* is the characteristic diffusion length (*i.e.* average FLG grain radius), 
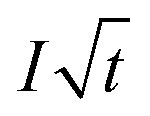
 is the Cottrell slope of amperometric titration curve, Δ*Q*(*E*)/Δ*E* is the charge at each potential step.

### Computational methods

We used the plane-wave density functional theory (DFT code Vienna *Ab initio* Simulation Package (VASP)^37,38^ to calculate the structural, electronic, and thermodynamic properties of alkali ion intercalated/co-intercalated FLG anodes. The generalized gradient approximation of DFT as proposed by Perdew, Burke, and Ernzerhof (GGA-PBE)^39^ that includes the spin-polarization effects was used. The core electrons and ion–electron interactions were treated by the projector-augmented wave (PAW) pseudopotentials methods.^40^ A plane wave cutoff of 550 eV, energy convergence criterion of 10^−4^ eV, and a force convergence criterion of 0.01 eV were utilized for the geometry optimizations. Grimme's empirical dispersion correction scheme (D3 method)^41^ was incorporated with the DFT calculations to account for the long-range effects in geometries and thermodynamic properties.

Because the FLG sample used in the electrochemical experiments contained ∼12–18 layers of graphene, we used a bulk graphene model in the calculations except for a few specific benchmark studies. Previous theoretical studies have shown that the computational setups employed here are suitable for the calculation of thermodynamics of alkali ion insertion in graphitic materials with good accuracy.^10,42,43^

When only a single-type of M is intercalated in FLG, the binding energy per metal (M = Li, K) atom is calculated by eqn (4).4
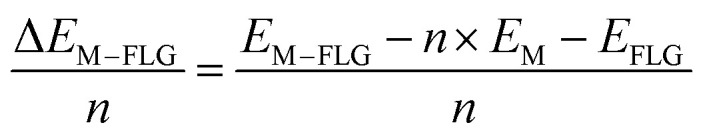
where Δ*E*_M–FLG_/*n* represents the binding energy (BE) per alkali atom (M) for the M intercalated FLG. *E*_M–FLG_, *E*_FLG_, and *E*_M_ are the energies of metal intercalated FLG, the free FLG, and energy of a single metal atom in the bulk form, respectively. Similarly, the average metal intercalation BE for FLGs with the co-intercalation of two types of M atoms is calculated using eqn (5).5
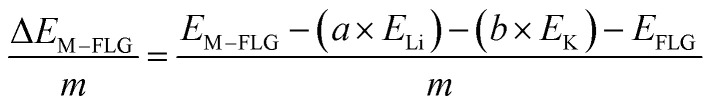
where, *m* = *a* + *b*; *a*, *b* = number of Li, K ions intercalated, respectively. *E*_Li_ and *E*_K_ are the energies of a single Li and K atom in the bulk metal, respectively.

## Results and discussion

### FLG characterization

**Fig. 1:**
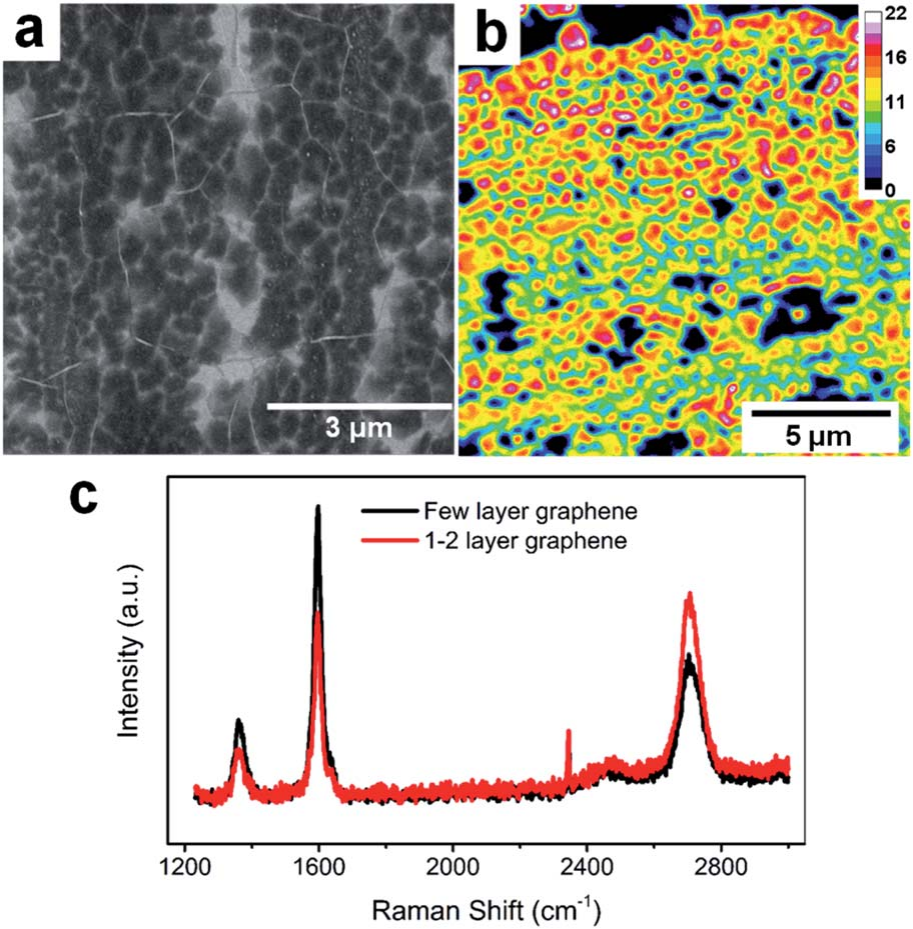
FLG electrode characterization. (a) SEM image of FLG. (b) FLG layer number distributions. (c) Raman spectra of thick and thinner graphene areas.

The FLG electrodes were grown using the CVD technique with a recipe from our previous report.^16^Fig. 1a is the SEM of our FLG graphene sample. Overall, the CVD grown FLG yields large area continuous sheet with polycrystalline nature, with 0.133 ± 0.064 μm^2^ average grain size and 206 ± 50 nm radius. Based on the calculated FLG layer number distribution obtained *via* optical transmittance microscopy, the majority of FLG grains consist of 12–18 layers graphene stacks over a continuous graphene sheet of 1–2 layers thick (Fig. 1b). This thickness distribution was further validated *via* Raman scattering (Fig. 1c), where the thicker grains have a Raman G/2D ratio larger than 1 (black trace), while thinner graphene regions reveal a characteristic G/2D ratio less than 1 (red trace).^44^ Overall, the thicker FLG grains serve as host of alkali ions while the thinner graphene serves as conductive base to collect current generated due to the (de)intercalation processes, making them good candidate platforms for fast analysis of Li^+^ intercalation and Li^+^/K^+^ co-intercalation processes.

### Li^+^ intercalation kinetics

We conducted a Nernstian-slope analysis to determine the number of charges transferred during Li^+^ (de)intercalation in FLG, by testing the Li^+^ insertion CV profile at various concentrations. In order to accurately obtain the Nernstian-slope, we prepared Li^+^-containing solutions in which concentrations (*C*_Li_) differed by 2 order of magnitude in the range between 0.6 mM and 0.1 M. To ease the quantification of the Nernstian slope, “*p*Li” (logarithm of the reciprocal of *C*_Li_, as an approximation to the ion activity) was applied as a numerical measure of the overall Li^+^ content, hence above solutions equivalent to *p*Li range of 3.2 to 1.0.

**Fig. 2:**
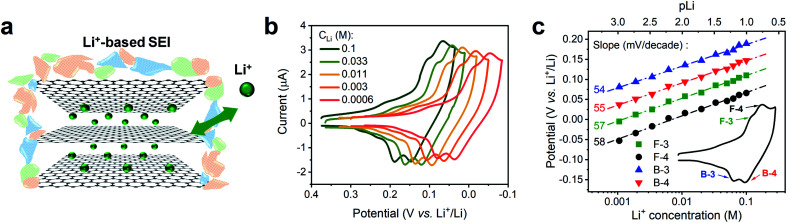
Voltametric characterization of Li^+^ intercalation on FLG at various concentrations. (a) Depiction of Li^+^ intercalation process in FLG with a Li^+^-based SEI. (b) Comparison of Li^+^ intercalation CVs at selected Li^+^ concentration (*C*_Li_). (c) Relationship of Li^+^ intercalation peak potential and *C*_Li_ at logarithmic scale (*p*Li) in panel (b) with labeled Nernstian-slopes, the inset CV indicates the selected peaks, F = forward/intercalation & B = backward/deintercalation. Li^+^ intercalations at various *C*_Li_ were tested by spiking different amount of 0.1 M TBAPF_6_ into 0.1 M LiPF_6_ PC–EC solution, on 7.1 mm^2^ FLG working electrode at 1 mV s^−1^.

The commonly used salt in organic carbonates, TBAPF_6_, was chosen as supporting electrolyte to reduce the resistance and balance the charge migration. Alkyl ammonium cations are known to easily intercalate and exfoliate graphite at less negative potentials than Li^+^.^45^ In our experiment, a preconditioned Li^+^-based SEI layer (Fig. 2a) was used to protect our FLG from the damage caused by TBA^+^ induced exfoliation.^45^ Previous work in our group has demonstrated that this preformed SEI layer can exclude the transportation of larger TBA^+^ through it, while allowing Li^+^ and K^+^ to diffuse at ease.^16^ The intercalation profiles at various *C*_Li_, 0.6 mM to 0.1 M, are shown in Fig. 2b. Each individual Li^+^ intercalation CV demonstrated similar staging-type signature with clearly defined (de)intercalation peaks compared to previous studies.^9,31^ Progressively negative (de)intercalation peak potential shifts were observed as *C*_Li_ decreased (*p*Li increased) (Fig. 2b). The robust CV shapes and current intensities evidenced the favorable FLG electrode condition was maintained throughout the tests (Fig. 2b). Hence these observed CV peak potential shifts directly represent the thermodynamic influence of *C*_Li_ on Li^+^ (de)intercalation.

The Li^+^ intercalation process in FLG can be simplified as eqn (6) below:

6 *x*Li^+^ + *x*e^−^ + *y*C = Li_*x*_C_*y*_

The Nernst equation of the above reaction at room temperature is:7
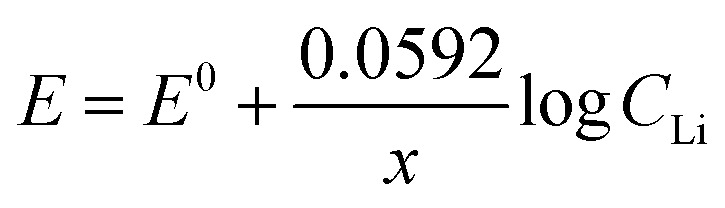


The value of Nernstian slope, 0.0592/*x*, can be used to calculate the number of electrons transferred (*x*) upon Li (de)intercalation. The relationships of peak potential and *C*_Li_ at logarithmic scale (*p*Li) were chosen to elucidate the number of Li^+^ transferred during Li^+^ (de)insertion processes (Fig. 2c), where the data was chosen from two pairs of representative (de)intercalation peaks as indicated in the figure inset. All peaks displayed a homogeneous linear relationship with Li^+^ concentration throughout the whole *C*_Li_ range, regardless of the phase transition between stages (Fig. S1a†). The Nernstian-slope of peak F-3, F-4, B-3, and B-4 were 57, 58, 54, and 55 mV per decade, respectively (Fig. 2c), which are all in close vicinity to 59 mV per decade for *x* = 1. We further carried out control experiments to unambiguously demonstrate that the observed shifts were not a product of potential drift at the reference electrode (Fig. S3†). Hence, we conclude that the Li^+^ (de)intercalation in FLG is a relatively isolated process, where the individual Li^+^ (de)insertion is not influenced by the surrounding Li^+^. Despite the complicity of multiple stage transition during Li^+^ (de)intercalation, it is not surprising to see only one Li^+^ participates at each step due to the relatively simple environment where only one type of ion intercalates.

### Experimental evidence of Li^+^ and K^+^ co-intercalation

**Fig. 3:**
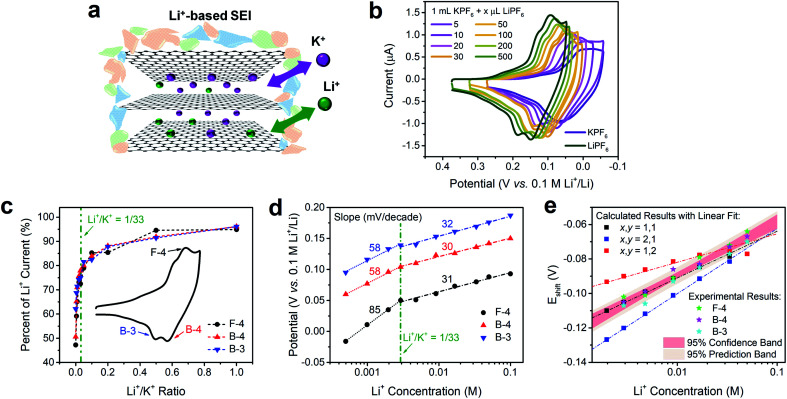
Voltametric characterization of intercalation on FLG in solutions containing both Li^+^ and K^+^ electrolytes. (a) Depiction of Li^+^/K^+^ co-intercalation process in FLG with a Li^+^-based SEI. (b) Comparison of intercalation CVs of Li^+^, K^+^ and selected Li^+^/K^+^ ratios. (c) Relationship of current percent change and Li^+^/K^+^ ratio from selected (de)intercalation peaks in panel (b). The percent changes were normalized to the pure Li^+^ intercalation peak (dark green trace in panel (b). (d) Relationship of Li^+^/K^+^ co-intercalation peak potential and Li^+^ concentration of selected (de)intercalation peaks in panel (b) with labeled Nernstian-slopes. (e) Comparison of calculated potential shift (*E*_shift_) and Li^+^ concentration relationship at various Li^+^ and K^+^ participation number (*x*,*y*), and their comparison with experimental *E*_shift_ at high Li^+^ concentrations. The 95% confidence and prediction bands come from the averaged results of experimental data. Li^+^/K^+^ co-intercalation relationship at various Li^+^/K^+^ ratio were tested by spiking different amounts of 0.1 M TBAPF_6_ into 0.1 M LiPF_6_ PC–EC solution, on 19.6 mm^2^ FLG working electrode at 500 μV s^−1^.

The intercalation behaviors of pure Li^+^ and K^+^ have been analyzed previously using CV and Raman spectroscopy.^9,16,46^ When the FLG sample surface is passivated by a fully-formed Li^+^-based SEI layer, both Li^+^ and K^+^ are able to intercalate in a facile and reversible electrochemical process (Fig. 3a).^16^ We note however, that there are similarities and differences between Li^+^ and K^+^ intercalation. Both Li^+^ and K^+^ have identical charge state and similar electrostatic interaction with graphene planes,^47^ and consequently fill the graphene interlayer spacing with the same staging-type order. Detailed DFT calculations on the thermodynamics of Li^+^ and K^+^ intercalation within a 4-layer graphene (4LG) at different stages and with different stoichiometries showed that both intercalation processes are thermodynamically favorable with clear staging behavior on FLG.^16,31^ However, the size difference between Li^+^ and K^+^ leads to stoichiometric change from LiC_6_ to KC_8_, thus resulting in a 1.33-fold decrease of the K^+^ intercalation charge when compared to its Li^+^ analogue.

In contrast, when Li^+^ and K^+^ co-existed in solution (Fig. 3a), we observed a distinct behavior that is not explained by the sum of individual intercalation processes. Increasing the concentration of Li^+^ into a K^+^ solution (Fig. 3b) leads to a continuous increase in peak currents and immediate positive shift in peak potentials of all intercalation and de-intercalation peaks. Using Li^+^ intercalation CV as reference (Fig. 3b, dark green trace), we obtained the normalized peak current changes at each Li^+^/K^+^ ratio. As shown in Fig. 3c, a sharp increase in the peak currents at low Li^+^ content was observed for three representative processes; an intercalation (F-4) and two deintercalation (B-3 and B-4), with a plateau behavior at the higher Li^+^/K^+^ ratios. One possible explanation of this phenomenon is the gradual stoichiometric transition of intercalated compounds with increasing Li^+^/K^+^ ratio, *e.g.*, changing from MC_8_ to MC_6_ (M = Li, K) for Stage 1 alkali intercalation. Furthermore, the potential of (de)intercalation peaks approached to pure Li^+^ intercalation case as well (Fig. 3d). Using Nernstian-relationship analysis, we noticed a progressive positive shift of the peak potentials as we increase the *C*_Li_. Compared to the monotonic changes in potential *vs.* Li^+^ concentration plot for pure Li^+^ intercalation case, the Li^+^/K^+^ co-intercalation system revealed two-stage incremental behavior with different slopes. Each addition of Li^+^ resulted in a positive shift which revealed two linear regimes, occurring at either Li^+^-rich or K^+^-rich conditions and denoted by the olive dashed vertical line in Fig. 3d. This transition between regimes happen at few mM of Li^+^ concentration, equivalent to a Li^+^/K^+^ atomic ratio of around 1/33 (Fig. S4†). These results were scan-rate independent as well (Fig. S5†), suggesting that these observations do not arise from kinetic or diffusion-related effects. With less than 5 mV shift of the Ag^+^/Ag reference at all test conditions (Fig. S3†), the influence of an unstable reference can be excluded as well.

**Fig. 4:**
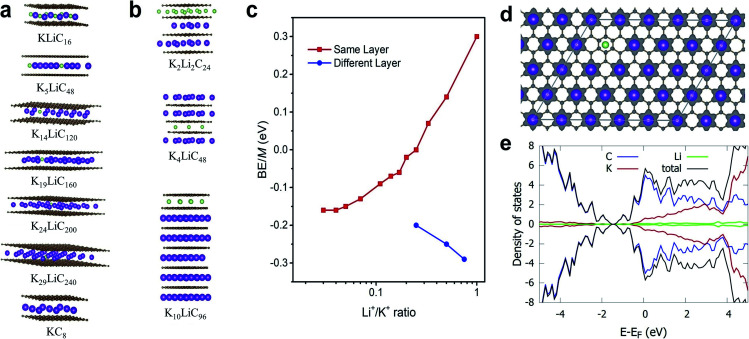
Different co-intercalation configurations and corresponding metal binding energies. (a and b) Selected models showing unique Li and K co-intercalation within the same or different layers, respectively. (c) Plot of the metal binding energies corresponding to co-intercalated metals ratio of Li/K (in a logarithmic scale) within the same or different layers, respectively. (d) Charge density difference plot in a section of K_19_C_160_Li. Only the positive isosurface is shown for clarity. (e) Projected density of states (pDOS) in K_29_LiC_240_. Colouring atom scheme: Li; green, K; purple.

Changes in Nernstian slopes between low and high Li^+^ concentration regimes revealed a strong sensitivity to the ratio of this ion (Fig. 3d). This is expected since the potential for Li^+^ intercalation is at least 100 mV more positive than K^+^ intercalation.^16^ Once spiked, even at low Li^+^ concentration, the thermodynamically more favorable Li^+^ insertion process starts participating in the bulk K^+^ dominated intercalation. In K^+^-rich regime, two deintercalation processes (Fig. 3d, B-3 and B-4 trace) revealed well-matched Nernstian-slopes of 58 mV per decade, indicating the single electron charge transfer processes during Li^+^ desertion. As a comparison, when the Li^+^ content in Li^+^/K^+^ mixture reached a certain threshold, *i.e.* 1/33 Li^+^/K^+^ ratio, the Nernstian-slope between apparent peak potentials and Li^+^ concentration for both intercalation and deintercalation process is reduced to *ca.* 30 mV per decade. With the Nernstian-slope decreased to half, it is evident that the Li^+^ intercalation mechanism has changed, from an independent Li^+^ intercalation at low Li^+^/K^+^ ratio regime (less than 1/33) to a Li^+^/K^+^ co-intercalation mechanism at high Li^+^/K^+^ ratio regime (more than 1/33). The intercalation process at this dilute Li^+^ concentration (Fig. 3d, F-4 trace) showed an 85 mV per decade slope, which is larger than a Nernstian slope of 59 mV per decade for the transfer of a singly charged species. Super-Nernstian responses have been reported potentiometrically during the ion exchange process for divalent alkaline-earth ions on singly charged anionic sites on polymer membranes and explained using models involving phase boundary equilibria.^48^ In our case, it is possible that simultaneous charging of Li^+^ and K^+^ on the graphene host, which creates distinct LiC_6_ and KC_8_ stoichiometries, leads also to structural or charge imbalances that widen the expected Nernstian response.

One possible explanation for the change of Nernstian slope at low/high Li^+^ concentration region is the potential cooperative co-intercalation process of Li^+^ and K^+^ with multiple electron transfer, as indicated by eqn (8) below:

8 *x*Li^+^ + *y*K^+^ + (*x* + *y*)e^−^ + *z*C = Li_*x*_K_*y*_C_*z*_

The Li^+^ and K^+^ coefficient values (*x*,*y*) will affect their (de)intercalation potentials, which can be rationalized in eqn (9), by the *E*_shift_*vs.* Li^+^ concentration trends (Fig. 3e) at various (*x*,*y*):9
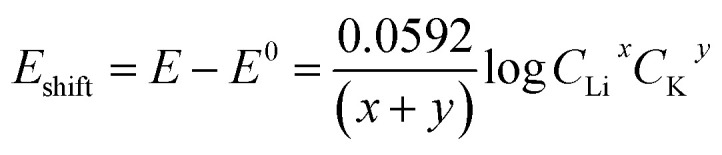


The detailed calculation procedure for theoretical co-intercalation *E*_shift_ and experimental *E*_shift_ can be found in the ESI (Tables S2 and S3†), where the theoretical *E*^0^ and experimental *E*^0^ at 1 M *C*_Li_ were used as inner reference points, respectively. The variation of Li^+^ and K^+^ participation in the co-intercalation reaction generated distinctive *E*_shift_ responses. Interestingly, the (*x*,*y*) = (1,1) co-intercalation case holds a Nernstian-slope of 26 mV per decade (Table S2†), which is similar to experimentally obtained results at high Li^+^ concentration (Fig. 3d). Furthermore, Fig. 3e (1,1) co-intercalation trace merged well with the experimental *E*_shift_ trend for all three representative peaks and their averaged result (Fig. S6†). Therefore, it is reasonable to assume that Li^+^ and K^+^ interact during the co-intercalation. In contrast to the work of Zheng *et al.* who reported a minor Li^+^ contribution on the K^+^ intercalation in a K_2_NiFe^II^(CN)_6_ cathode,^49^ our samples demonstrate participation from both ions in a mixed system. In addition, the facile transport of both alkali ions creates opportunities for concurrent insertion of both Li^+^ and K^+^ into graphene sheets. Nonetheless, questions emerge regarding the feasibility of K^+^ intercalation in the presence of a lithiated phase, as expected by a Li^+^ intercalation potential that is more positive. For this purpose, we turned to DFT methods to elucidate the energetics of this process.

### Theoretical mechanistic study of Li^+^ and K^+^ co-intercalation

Theoretically, both alkali ions can be intercalated either within the same or separate layers of the FLG. We performed DFT calculations to understand how Li^+^ and K^+^ would co-exist, either in the same layer or into separate layers. Specifically, we calculated the energetics corresponding to co-intercalated FLG systems with Li^+^ and K^+^ existing in a completely mixed form with random K–Li phases and for systems with separate Li^+^-only and K^+^-only domains (Fig. 4a–c). In the latter systems, large boundaries will separate the Li^+^ and K^+^ domains (Table 1). Depending on forward and reverse processes; *i.e.*, substituting Li^+^ by K^+^ in a completely Li intercalated system and the *vice versa*, these domains can be formed at different time scales. The calculations show that for a hypothetical co-intercalation system with 50% each Li^+^ and K^+^ intercalation with all –K–Li– phases, the system is thermodynamically unstable by 0.30 eV per atom. When the concentration of –K–Li– phases decreased for larger K^+^/Li^+^ ratio, the system becomes thermodynamically stable starting from K_5_LiC_48_. For K_29_LiC_240_ the energy gain due to intercalation is −0.16 eV per atom. This ratio is already close to the experimentally observed kinetic transition point at K_33_Li system (Fig. S4†). We also note that the fully potassiated KC_8_ has a calculated binding energy (BE) of −0.20 eV. These calculations clearly show that substituting Li^+^ for K^+^ within the same layer in a FLG is thermodynamically favorable when intercalated K^+^ and Li^+^ are separated as far as possible or when K^+^ only and Li^+^ only domains exist within the FLG layers. This situation arises due to the size-difference between Li^+^ and K^+^ ions, which will disfavor the simultaneous binding of both ions with graphene for –K–Li– phases. However, when Li^+^ and K^+^ domains could form at large Li^+^/K^+^ ratios, both ions can favorably interact with the graphene layers.

**Table 1:** DFT calculated energetics for K_*x*−1_LiC_8*x*_ type bulk models, in which the K^+^/Li^+^ ratio (*x* − 1 : 1) in any interlayer has been kept constant. For comparison, energies for LiC_8_ and KC_8_ are also presented

System	K^+^/Li^+^ intercalation patterns within the same layer	BE/M atom^a^, eV
LiC_8_	–Li–	−0.26
KLiC_16_	–K–Li–	0.30
K_2_LiC_24_	–K_2_–Li–	0.14
K_3_LiC_32_	–K_3_–Li–	0.07
K_4_LiC_40_	–K_4_–Li–	0.00
K_5_LiC_48_	–K_5_–Li–	−0.02
K_6_LiC_56_	–K_6_–Li–	−0.06
K_7_LiC_64_	–K_7_–Li–	−0.07
K_9_LiC_80_	–K_9_–Li–	−0.09
K_14_LiC_120_	–K_14_–Li–	−0.13
K_19_LiC_160_	–K_19_–Li–	−0.15
K_24_LiC_200_	–K_24_–Li–	−0.16
K_29_LiC_240_	–K_29_–Li–	−0.16
KC_8_	–K–	−0.20

a w.r.to M atom in its stable crystal.

Co-intercalation at large Li^+^/K^+^ ratios were further investigated by modelling Li^+^ and K^+^ intercalation in separate layers of FLG (Fig. 4b). We note that using the model systems with Li^+^ and K^+^ intercalation in separate layers significantly reduce the computational time while replicating the effects of co-intercalation phases such as –Li_*a*_K_*b*_– (*a*, *b* > 33) in the same graphene layer. We found that the intercalation of Li^+^ and K^+^ in separate layers (or Li^+^-only and K^+^-only domains formation in same layer) is always thermodynamically stable. Li^+^-domains and K^+^-domains are expected to form LiC_6_- and KC_8_-type configurations to maximize the stability. In fact, the calculations predict that at 4 : 3 ratio of Li^+^/K^+^ ions, a Stage 1 co-intercalation configuration with both C_6_Li- and C_8_K-type stoichiometries could exist with a binding energy of −0.29 eV M^−1^, which is even more stable compared to individual C_8_Li and C_8_K intercalation systems. To summarize, the DFT calculations clearly showed that the co-intercalation is stable for a range of Li^+^/K^+^ ratios and explained the corresponding experimental findings. We note that in addition to the binding energies discussed here, the entropy could be important to understand certain features for staging mechanisms during Li ion intercalation.^50–52^ However, such contributions could be neglected safely with non-significant errors when computing the general thermodynamic properties of intercalation phenomena, especially at room temperature.^14,53^

DFT calculated average atomic charges on Li^+^ and K^+^ ions in co-intercalated systems (Table S4†) showed that the charge on K^+^ remains consistent (0.80–0.82*e*^−^) for all concentrations compared to a value of 0.82*e*^−^ in the bulk KC_8_. The magnitude of atomic charge on Li^+^ fluctuates from 0.84–0.91*e*^−^, depending on the size of –K–Li– phases. The maximum value of Li^+^ charge (0.91*e*^−^) was predicted for thermodynamically unstable KLiC_16_ system. For larger K^+^/Li^+^ ratio, the charge on Li^+^ becomes consistent with the values of 0.84–0.86*e*^−^ compared to 0.84*e*^−^ in bulk LiC_8_. Overall, both alkali ions exist in monovalent ionic forms in all co-intercalation systems studied. Calculated charge density difference plot of co-intercalation systems suggest stronger charge transfer interactions of the graphene layers with K^+^ than Li^+^ when the –K–Li– phases exist in the same layer (Fig. 4d). This support our prediction that the K^+^ and Li^+^ will likely form separate domains in co-intercalation systems. Calculated projected DOSs demonstrated the metallic nature of the co-intercalation systems and substantiated the conclusions drawn from the calculated atomic charges and charge density plots (Fig. 4e).

### Diffusion coefficient for co-intercalation

We note that the change of external environment, *i.e.*, Li^+^/K^+^ ratio, may also cause changes in the apparent diffusion coefficient of alkali ions at each intercalation stage. During (de)insertion, the Li^+^ and K^+^ are constrained within the gap between two graphene planes. The larger K^+^ (1.4 Å) leads to a larger expansion of the graphene interlayer distance, from 3.35 Å to 5.32 Å at Stage 1 KC_8_ compound, while the smaller Li^+^ (0.76 Å) leads to a smaller expansion of 3.61 Å at Stage 1 LiC_6_ compound.^54^ Hence, it is possible that the Li^+^ and K^+^ exhibit different diffusion rates within graphene planes. Thus, we used the potential intermittent titration technique (PITT) to examine the apparent diffusion coefficient in various Li^+^ and K^+^ containing solutions.^35^

**Fig. 5:**
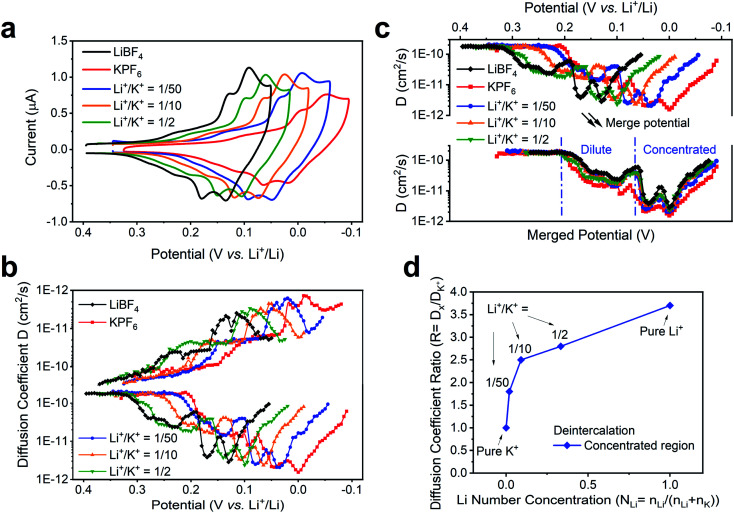
Diffusion coefficient of co-intercalation system. (a) Li^+^, K^+^ intercalation and co-intercalation behavior at various ratio. (b) Diffusion coefficients of alkali ions at various Li^+^ and K^+^ ratio. The value of diffusion coefficients was obtained *via* PITT analysis at different Li^+^, K^+^ solution mixture. (c) Illustration of the method of merge potentials of different responses in Li^+^/K^+^ co-intercalation systems for diffusion coefficient comparison. We defined here the potential range for the “dilute” and “concentrated” regions for future calculation. (d) The ratio of diffusion coefficient of deintercalation at concentrated region *vs.* that of the pure K^+^ system. Diffusion coefficient ratio *R* = *D*_*x*_/*D*_K_; Li number concentration *N*_Li_ = *n*_Li_/(*n*_Li_ + *n*_K_). All experiments were tested in 0.1 M LiBF_4_, KPF_6_ or LiPF_6_ in PC–EC, on 4.9 mm^2^ FLG working electrode at 1 mV s^−1^.

Fig. 5a displays the CV of conditions chosen to measure diffusion coefficients. In agreement with the previously determined voltammetric peak displacements (Fig. 3b), similar positive potential shift upon addition of Li^+^ into bulk K^+^ electrolyte is observed (Fig. 5a). The calculated diffusion coefficients at each potential were also plotted in Fig. 5b. Overall, the apparent diffusion coefficient demonstrated a stage-dependent behavior with values in the range of 1 × 10^−10^ to 1 × 10^−12^ cm^2^ s^−1^, which is comparable with previous reports of alkali ion insertion in graphite.^55,56^ For comparison purposes, the diffusion coefficient distribution at different Li^+^/K^+^ co-intercalation conditions was overlaid by properly shifting their potentials (Fig. 5c). While all co-intercalation systems held similar staging-type behavior of the apparent diffusion coefficient, the earlier stages (FLG to Stage 3) exhibited 1 order of magnitude faster ionic diffusion than later stages (Stage 3 to Stage 1). This can be explained by the concentration difference of intercalated alkali ions inside FLG. This is, as more alkali ions inserted within graphene planes, the scattering of newly intercalated alkali ions increases, hence reducing the mean free path of intercalated ions. Therefore, the dilute region has relatively larger diffusion coefficient than the concentrated region, as shown in Fig. 5c. The values of the average diffusion coefficient in the dilute and concentrated regions can be found in Table S5.†

The diffusion coefficients in co-intercalation systems also revealed a component-dependent behavior. As shown in Fig. 5b and Table S4,† increasing Li^+^ content leads to larger apparent diffusion coefficient. In fact, the pristine K^+^ (Fig. 5b red-trace) diffuses ∼3.2 times slower than pristine Li^+^ case (Fig. 5b black-trace), which correlates with the size difference between smaller Li^+^ and larger K^+^. As presented in the overlaid results (Fig. 5c), all co-intercalation systems followed similar stage-dependent distributions but have different diffusion coefficient values. Therefore, we calculated the point-to-point diffusion coefficient ratio of the experimentally determined diffusion coefficient with respect to that of the pure K^+^ system (Table S4†) and plotted the trends of all cases and selected representative condition in Fig. S7† and 5d. A sample data analysis of *D*_Li_^+^/*D*_K_^+^ can be found in Fig. S8.† We observed an increasing trend of the *D*_*x*_/*D*_K_ ratio as the Li^+^ content was increased for both intercalation and deintercalation processes (Fig. 5d). This suggests a strong dependence of the species mobility within the host as a function of electrolyte composition, thus supporting the hypothesis that co-intercalation takes places. Furthermore, the highest changes were observed as the composition of the electrolyte departed from pure K^+^ to Li^+^/K^+^ = 1/50, and before the observed transition occurring at ∼1/33 Li^+^/K^+^ in Fig. 3d. The ratio in remaining regions scales almost linearly with Li^+^ number concentration (Fig. 5d). The result can be explained by the changes of co-intercalation dynamics (Fig. 4c), where the co-intercalation feature transitions from a mixed component structure (Fig. 4a) to layered one (Fig. 4b), thus altering the diffusion coefficient response as the alkali ion component changes.

## Conclusion

Facile K^+^ and Li^+^ (de)intercalation is possible through formation of a stable SEI capable of yielding high quality CVs that were analyzed to determine the current–potential relations and further to determine diffusion coefficients within the host. Using this versatile electrochemical inspection of FLG electrodes, we determined for the first time that Li^+^ (de)intercalation follows a Nernstian behavior, with staging voltammetric signatures displaying slopes *ca.* 54–58 mV per decade. Furthermore, the impact of alkali ion electrolyte composition on the co-intercalation behavior for mixtures of electrochemically reversible Li^+^ and K^+^ on a well-defined FLG carbon electrode was explored. The analysis of CVs at different Li^+^/K^+^ concentration ratios suggested two different co-intercalation regimes with a transition at approximately 3% Li^+^/K^+^ ratio. In the Li^+^/K^+^ mixture, at K^+^-rich regime, the Li^+^ intercalate independently; at Li^+^-rich regime, the Li^+^ and K^+^ co-intercalation at approximately 1 to 1 ratio. These findings were rationalized by performing DFT simulations of Li^+^ and K^+^ intercalation, as well as by considerations of the different intercalation potentials for these ions. Consistent with experimental data, the DFT simulations found that Li^+^ intercalation is more energetically favorable than K^+^ intercalation. DFT also suggested that the co-intercalation of K^+^ in Li^+^ rich conditions was more energetically favorable than Li^+^ intercalation in K^+^-rich conditions, although both cases were more favorable than pure K^+^ intercalation. These results explain the preference for Li^+^ over K^+^ during staging-type intercalation, but also establish the feasibility of co-intercalation. Further, we observed differences in the diffusion coefficients for the ions within the host as a function of electrolyte composition, which supports the hypothesis of a co-intercalated system. While previous studies of co-intercalation on graphitic anode typically focus on co-intercalation of solvents and one particular alkali ion,^20^ this is to the best of our knowledge the first study elucidating the intercalation behavior of two monovalent alkali ions. By exploring Li^+^ and K^+^ co-intercalation through an experimental and theoretical framework, this work provides a better fundamental electrochemical approach to understand co-intercalation processes. Intercalating two different ions simultaneously and controlling this process by means of the solution composition opens exciting new directions for energy storage, since potentially new ion combinations such as those that are energetically unfavorable (*e.g*. Na^+^)^42^ could be paired with reversible ones (*e.g.* Li^+^ and K^+^) to access their electrochemistry. Research towards that objective is currently in progress at our laboratories.

## Conflicts of interest

The authors declare no competing financial interest.

## Supplementary Material

SC-012-D0SC03226C-s001

## References

[cit1] Nitta N., Wu F., Lee J. T., Yushin G. (2015). Mater. Today.

[cit2] Eftekhari A., Jian Z. L., Ji X. L. (2017). ACS Appl. Mater. Interfaces.

[cit3] Katorova N. S., Fedotov S. S., Rupasov D. P., Luchinin N. D., Delattre B., Chiang Y.-M., Abakumov A. M., Stevenson K. J. (2019). ACS Appl. Energy Mater..

[cit4] Zhang H., Zhao H., Khan M. A., Zou W., Xu J., Zhang L., Zhang J. (2018). J. Mater. Chem. A.

[cit5] Dahn J. R. (1991). Phys. Rev. B: Condens. Matter Mater. Phys..

[cit6] Aurbach D. (2000). J. Power Sources.

[cit7] Ohzuku T., Iwakoshi Y., Sawai K. (1993). J. Electrochem. Soc..

[cit8] Xu K. (2014). Chem. Rev..

[cit9] Levi M. D., Aurbach D. (1997). J. Electroanal. Chem..

[cit10] Jian Z. L., Luo W., Ji X. L. (2015). J. Am. Chem. Soc..

[cit11] Levi M. D., Levi E., Aurbach D., Schmidt M., Oesten R., Heider U. (2001). J. Power Sources.

[cit12] Levi M. D., Levi E. A., Aurbach D. (1997). J. Electroanal. Chem..

[cit13] Shi Q., Dokko K., Scherson D. A. (2004). J. Phys. Chem. B.

[cit14] Li Y., Lu Y., Adelhelm P., Titirici M.-M., Hu Y.-S. (2019). Chem. Soc. Rev..

[cit15] Boesenberg U., Sokaras D., Nordlund D., Weng T.-C., Gorelov E., Richardson T. J., Kostecki R., Cabana J. (2019). Carbon.

[cit16] Hui J., Schorr N. B., Pakhira S., Qu Z., Mendoza-Cortes J. L., Rodríguez-López J. (2018). J. Am. Chem. Soc..

[cit17] Kim H., Hong J., Yoon G., Kim H., Park K.-Y., Park M.-S., Yoon W.-S., Kang K. (2015). Energy Environ. Sci..

[cit18] Katorova N. S., Luchkin S. Y., Rupasov D. P., Abakumov A. M., Stevenson K. J. (2020). J. Chem. Phys..

[cit19] Cohn A. P., Muralidharan N., Carter R., Share K., Oakes L., Pint C. L. (2016). J. Mater. Chem. A.

[cit20] Cohn A. P., Share K., Carter R., Oakes L., Pint C. L. (2016). Nano Lett..

[cit21] Kim H., Yoon G., Lim K. M., Kang K. (2016). Chem. Commun..

[cit22] Moon H., Tatara R., Mandai T., Ueno K., Yoshida K., Tachikawa N., Yasuda T., Dokko K., Watanabe M. (2014). J. Phys. Chem. C.

[cit23] Wu N., Yang Z. Z., Yao H. R., Yin Y. X., Gu L., Guo Y. G. (2015). Angew. Chem., Int. Ed..

[cit24] Wang Y., Wang C., Yi X., Hu Y., Wang L., Ma L., Zhu G., Chen T., Jin Z. (2019). Energy Storage Materials.

[cit25] Gao Q., Come J., Naguib M., Jesse S., Gogotsi Y., Balke N. (2017). Faraday Discuss..

[cit26] Liu F., Liu Y., Zhao X., Liu K., Yin H., Fan L.-Z. (2020). Small.

[cit27] Rojas-Carbonell S., Artyushkova K., Serov A., Santoro C., Matanovic I., Atanassov P. (2018). ACS Catal..

[cit28] Walczak M. M., Dryer D. A., Jacobson D. D., Foss M. G., Flynn N. T. (1997). J. Chem. Educ..

[cit29] Zhang Q., Guo Q., White R. E. (2006). J. Electrochem. Soc..

[cit30] Guo Q., White R. E. (2005). J. Electrochem. Soc..

[cit31] Hui J., Burgess M., Zhang J., Rodríguez-López J. (2016). ACS Nano.

[cit32] Levi M. D., Aurbach D. (1997). J. Phys. Chem. B.

[cit33] Levi M. D., Aurbach D. (2012). Charact. Mater..

[cit34] Nair R. R., Blake P., Grigorenko A. N., Novoselov K. S., Booth T. J., Stauber T., Peres N. M. R., Geim A. K. (2008). Science.

[cit35] Hui J., Gossage Z. T., Sarbapalli D., Hernández-Burgos K., Rodríguez-López J. (2019). Anal. Chem..

[cit36] Kaspar J., Graczyk-Zajac M., Riedel R. (2014). Electrochim. Acta.

[cit37] Kresse G., Furthmüller J. (1996). Phys. Rev. B: Condens. Matter Mater. Phys..

[cit38] Kresse G., Hafner J. (1993). Phys. Rev. B: Condens. Matter Mater. Phys..

[cit39] Perdew J. P., Burke K., Ernzerhof M. (1996). Phys. Rev. Lett..

[cit40] Blöchl P. E. (1994). Phys. Rev. B: Condens. Matter Mater. Phys..

[cit41] Grimme S., Antony J., Ehrlich S., Krieg H. (2010). J. Chem. Phys..

[cit42] Liu Y., Merinov B. V., Goddard W. A. (2016). Proc. Natl. Acad. Sci. U.S.A..

[cit43] Hazrati E., de Wijs G. A., Brocks G. (2014). Phys. Rev. B: Condens. Matter Mater. Phys..

[cit44] Graf D., Molitor F., Ensslin K., Stampfer C., Jungen A., Hierold C., Wirtz L. (2007). Nano Lett..

[cit45] Cooper A. J., Wilson N. R., Kinloch I. A., Dryfe R. A. W. (2014). Carbon.

[cit46] Zou J., Sole C., Drewett N. E., Velický M., Hardwick L. J. (2016). J. Phys. Chem. Lett..

[cit47] Dresselhaus M. S., Dresselhaus G. (2002). Adv. Phys..

[cit48] Amemiya S., Bühlmann P., Umezawa Y. (1998). Anal. Chem..

[cit49] Zheng J., Deng W., Hu Z., Zhuo Z., Liu F., Chen H., Lin Y., Yang W., Amine K., Li R., Lu J., Pan F. (2018). ACS Energy Lett.

[cit50] Leiva E. P. M., Perassi E., Barraco D. (2016). J. Electrochem. Soc..

[cit51] Otero M., Sigal A., Perassi E. M., Barraco D., Leiva E. P. M. (2017). Electrochim. Acta.

[cit52] Yazami R., Reynier Y. (2006). J. Power Sources.

[cit53] Raju M., Ganesh P., Kent P. R. C., van Duin A. C. T. (2015). J. Chem. Theory Comput..

[cit54] Nijamudheen A., Sarbapalli D., Hui J., Rodríguez-López J., Mendoza-Cortes J. L. (2020). ACS Appl. Mater. Interfaces.

[cit55] Levi M. D., Aurbach D. (1997). J. Phys. Chem. B.

[cit56] Persson K., Sethuraman V. A., Hardwick L. J., Hinuma Y., Meng Y. S., van der Ven A., Srinivasan V., Kostecki R., Ceder G. (2010). J. Phys. Chem. Lett..

