# Supplementary material for: Nernstian Li+ intercalation into few-layer graphene and its use for the determination of K+ co-intercalation processes
Source: Chem Sci. 2020 Oct 8;12(2):559–68. doi: 10.1039/d0sc03226c (PMC8179004; doi:10.1039/d0sc03226c)
Supplement: SC-012-D0SC03226C-s001 [file SC-012-D0SC03226C-s001.pdf]

Electronic Supplementary Material (ESI) for Chemical Science.
This journal is © The Royal Society of Chemistry 2020

Supporting Information

Nernstian Li^+ Intercalation into Few-Layer Graphene and its Use for Determination of K^+ Co-Intercalation Processes

Jingshu Hui,[a,#] A. Nijamudheen,,[b,c,#] Dipobrato Sarbapalli,[e] Chang Xia,[a] Zihan Qu,[a] Jose L. Mendoza-Cortes,[b,d,] and Joaquín Rodríguez-López[a,]**

[a]Department of Chemistry, University of Illinois at Urbana–Champaign, 600 South Mathews Avenue, Urbana, Illinois 61801, United States

[b]Department of Chemical & Biomedical Engineering, Florida A&M – Florida State University, Joint College of Engineering, 2525 Pottsdamer Street, Tallahassee, Florida, 32310, United States

[c]*Current Address*: Chemistry Division, Brookhaven National Laboratory, Upton, New York 11973-5000, United States.

[d]*Current Address*: Department of Chemical Engineering & Materials Science, Michigan State University, East Lansing, Michigan 48824, United States.

[e]Department of Materials Science and Engineering, University of Illinois at Urbana−Champaign, 1304 West Green Street, Urbana, Illinois 61801, United States.

*To whom all correspondence should be addressed:

Prof. Mendoza-Cortes: jmendoza@msu.edu (Email) and 517-355-5135 (Phone)

Prof. Rodríguez-López: joaquinr@illinois.edu (Email) and 217-300-7354 (Phone)

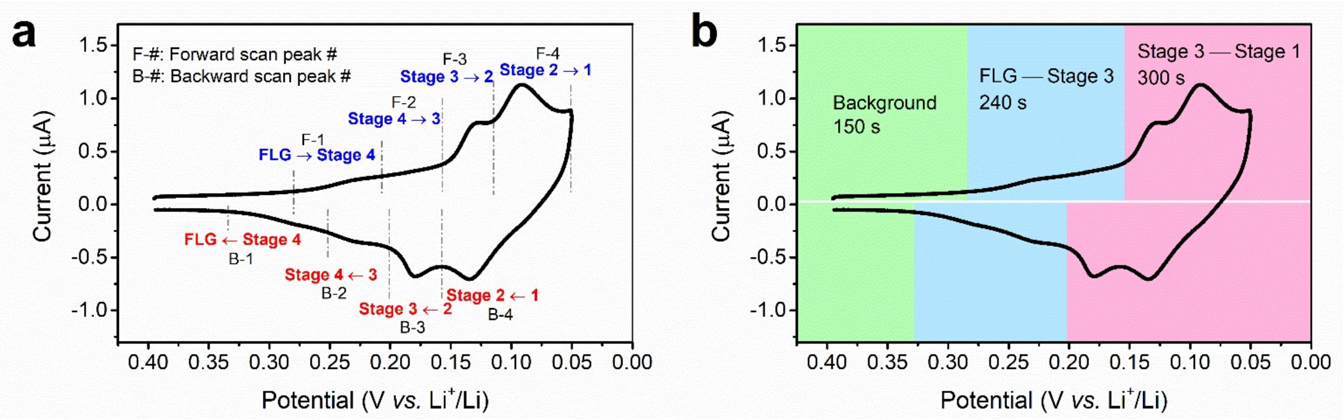

Figure S1. Intercalation stages notation and PITT titration duration. **a**, Demonstration of position of various alkali ion intercalation stages and their peak notations used in main text. **b**, Based on the level of current, experimental PITT titration duration were applied as 150 s, 240 s, and 300 s for background, FLG to Stage 3, and Stage 3 to Stage 1 regions, respectively.

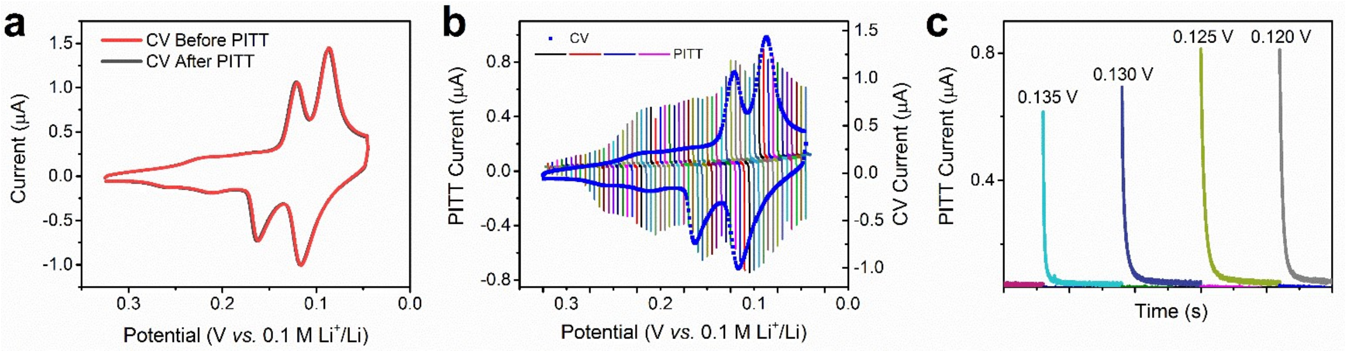

Figure S2. Demonstration of PITT method. **a**, CV of FLG before and after PITT test. **b**, Comparison of PITT current-potential transient relationship and the CV behavior of FLG. **c**, Zoom in of PITT result showing individual titration curves.

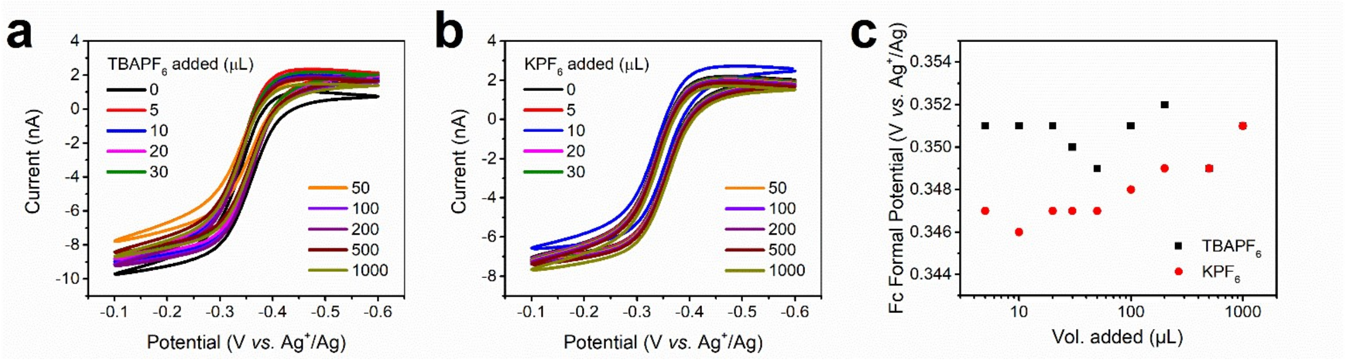

Figure S3. Characterization of Ag^{+}/Ag reference stability. **a**, Ferrocene (Fc) CVs on 12.5 µm Pt tip working electrode in different ratio Li^{+}/TBA^{+} mixtures. The amount of 0.1 M $TBAPF_6$ added to 1 mL of 0.1 M $LiPF_6$ were indicated in the figure's caption. **b**, Fc CVs on 12.5 µm Pt tip in different ratio Li^{+}/K^{+} mixtures. The amount of 0.1 M KPF_6 added to 1 mL of 0.1 M $LiPF_6$ were indicated in the figure's caption. **c**, Summary of Fc's formal potential with total amount of $TBAPF_6$ or KPF_6 added, showing less than 5 mV reference potential shift throughout the whole range. Ferrocene CVs were obtained at scan rate of 50 mVs^{-1}.

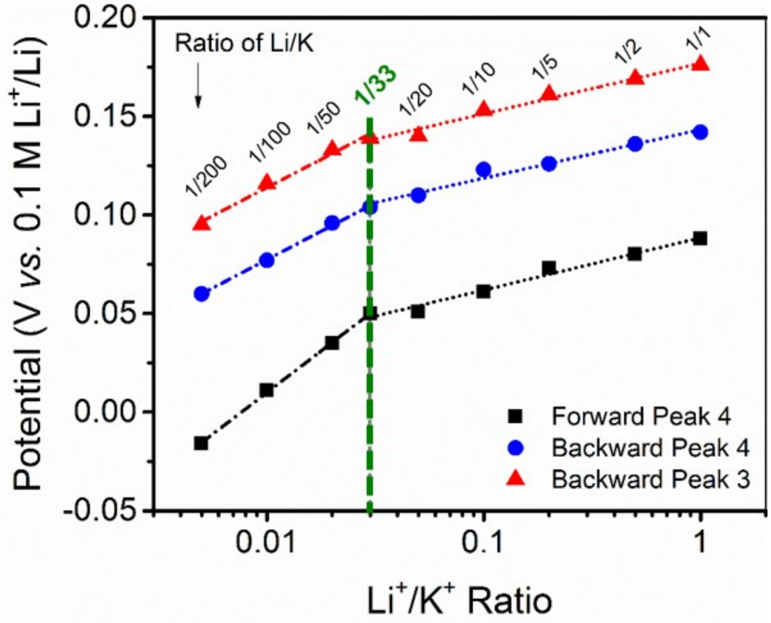

Figure S4. Relationship of peak potential and Li^+/K^+ Ratio of selected (de-)intercalation peaks in Figure 3d.

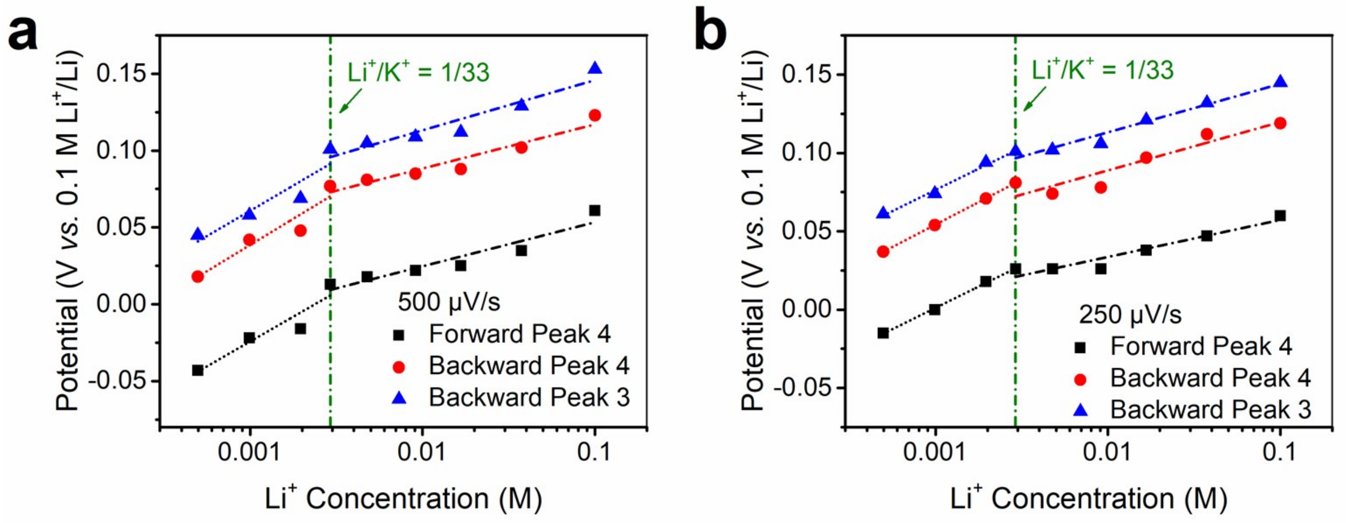

Figure S5. Relationship of peak potential and Li^+ concentration of (de-)intercalation peaks at different Li^+ concentration with a constant 0.1 M KPF_6 concentration. All solutions are 0.1 M $LiBF_4$, KPF_6 or $LiPF_6$ in PC-EC, scan rates are 500 µVs^{-1} (panel **a**) and 250 µVs^{-1} (panel **b**).

Table S1. Fitting results of Figure 3d and Figure S5.

		4th Intercalation	3rd Deintercalation	4th Deintercalation
Slope of region-I [a] / mV	500 µVs^{-1} (Figure 3d)	85	58	58
	500 µVs^{-1} (Figure S5a)	65	66	68
	250 µVs^{-1} (Figure S5b)	54	54	57
Slope of region-II [b] / mV	500 µVs^{-1} (Figure 3d)	31	32	30
	500 µVs^{-1} (Figure S5a)	29	33	29
	250 µVs^{-1} (Figure S5b)	28	31	31

[a] Region-I: 0 to 2.9 mM Li^+.
[b] Region-II: 2.9 mM to 0.1 M Li^+.

Calculation of Potential Shift in Li^+/K^+ Co-intercalation at Various Conditions

$$x\mathrm{Li}^+ + y\mathrm{K}^+ + (x+y)e^- + z\mathrm{C} = \mathrm{Li}_x\mathrm{K}_y\mathrm{C}_z \tag{6}$$

$$E = E^o + \frac{0.0592}{(x+y)} log C_{Li}{}^x C_K{}^y \tag{S1}$$

$$E_{shift} = E - E^o = \frac{0.0592}{(x+y)} log C_{Li}{}^x C_K{}^y \tag{7}$$

Table S2. Calculated E_{shift} and Nernstian-slope for Li^+/K^+ Co-intercalation

C_{Li} (M)	C_K (M)	E_{shift} (V)		
		x,y = 1,1	x,y = 1,2	x,y = 2,1
4.98E-04	9.95E-02	-0.127	-0.105	-0.150
9.90E-04	9.90E-02	-0.119	-0.099	-0.138
1.96E-03	9.80E-02	-0.110	-0.093	-0.127
2.91E-03	9.71E-02	-0.105	-0.090	-0.120
4.76E-03	9.52E-02	-0.099	-0.086	-0.112
9.09E-03	9.09E-02	-0.091	-0.081	-0.101
1.67E-02	8.33E-02	-0.085	-0.078	-0.091
3.33E-02	6.67E-02	-0.079	-0.076	-0.082
5.00E-02	5.00E-02	-0.077	-0.077	-0.077
Slope_E_{shift} *vs.* Log C_{Li} (mV/decade)		26	15	37
R^2		0.991	0.955	0.998

Calculation of experimental E_{shift} in Figure 3e

The experimental potential shift calculation applied equation S1 below. Noticing the potential of corresponding Li^+ intercalation peak at C_{Li} = 1 M were referred as inner reference for the E_{shift} calculation:

$$E_{shift} = E_{expt} - E_{CLi\,=\,1\,M} \qquad \text{(S2)}$$

In experiments (Figure 3b), maximum concentration of C_{Li} = 0.1 M (C_K = 0 M) was tested. The $E_{CLi = 1 M}$ were calculated based on equation S2 below:

$$E_{CLi\,=\,1\,M} = E_{CLi\,=\,0.1\,M} + 0.059\,V \qquad \text{(S3)}$$

The calculated result is shown in Table S3 and Figure S6 below.

Table S3. Calculated experimental E_{shift} in Figure 3e and their Nernstian-slopes.

C_{Li} (M)	Experimental Potential (V *vs.* Li^+/Li)			E_{shift} (V)			
	F-4	B-4	B-3	F-4	B-4	B-3	Average
2.91E-03	0.05	0.104	0.139	-0.102	-0.105	-0.107	-0.105
4.76E-03	0.051	0.11	0.14	-0.101	-0.099	-0.106	-0.102
9.09E-03	0.061	0.123	0.153	-0.091	-0.086	-0.093	-0.090
1.67E-02	0.073	0.126	0.161	-0.079	-0.083	-0.085	-0.082
3.33E-02	0.08	0.136	0.169	-0.072	-0.073	-0.077	-0.074
5.00E-02	0.088	0.142	0.176	-0.064	-0.067	-0.070	-0.067
1.00E-01	0.093	0.15	0.187				
1.00[a]	0.152	0.209	0.246				
Slope_E_{shift} *vs.* Log C_{Li} (mV/decade)				31	30	32	31
R^2				0.979	0.985	0.984	0.991

[a] Calculation based on equation S2.

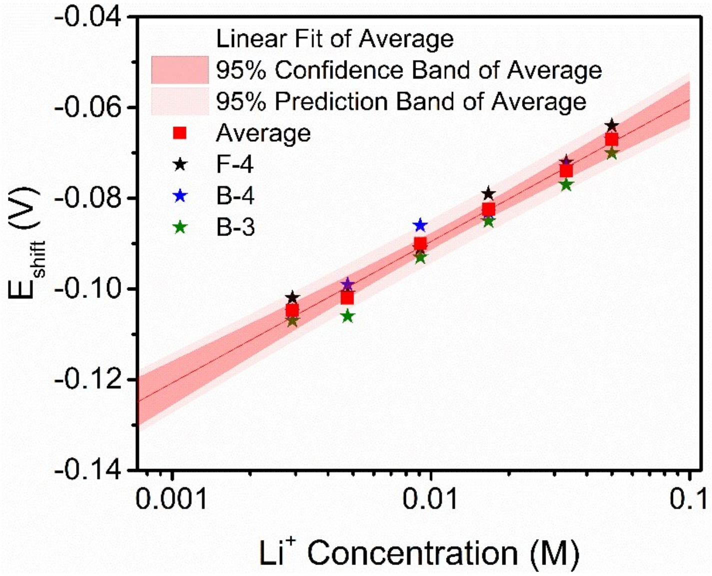

Figure S6. Comparison of calculated experimental E_{shift} and their averages. The E_{shift} of three experimental representative peaks (F-4, B-4, B-3) and their averages are plotted here. The red line and shadowed area indicate the linear regression fitting and 95% Confidence and Prediction band of the average E_{shift}.

Table S4. DFT calculated average atomic charges of co-intercalated Li^+ and K^+ ions.

System	K^+/Li^+ intercalation patterns within the same layer	BE/M atom, eV[a]	Average atomic charge	
			K^+	Li^+
LiC_8	-Li-	-0.26	-	0.84
$KLiC_{16}$	-K-Li-	0.30	0.80	0.91
K_2LiC_{24}	$-K_2$-Li-	0.14	0.81	0.89
K_3LiC_{32}	$-K_3$-Li-	0.07	0.81	0.85
K_4LiC_{40}	$-K_4$-Li-	0.00	0.82	0.88
K_5LiC_{48}	$-K_5$-Li-	-0.02	0.81	0.85
K_6LiC_{56}	$-K_6$-Li-	-0.06	0.81	0.87
K_7LiC_{64}	$-K_7$-Li-	-0.07	0.82	0.86
K_9LiC_{80}	$-K_9$-Li-	-0.09	0.82	0.86
$K_{14}LiC_{120}$	$-K_{14}$-Li-	-0.13	0.82	0.85
$K_{19}LiC_{160}$	$-K_{19}$-Li-	-0.15	0.82	0.86
$K_{24}LiC_{200}$	$-K_{24}$-Li-	-0.16	0.82	0.86
$K_{29}LiC_{240}$	$-K_{29}$-Li-	-0.16	-	-
KC_8	-K-	-0.20	0.82	-

[a] w.r.t. Alkali atom (M) in its stable crystal

Table S5. Diffusion coefficient analysis of Li^+/K^+ co-intercalation

	Conditions	Dilute[a]	Concentrated[a]	Total[b]
**Intercalation**				
Average diffusion coefficient D (cm^2/s)	K^+	1.9 E-11 ± 4.3 E-12	3.8 E-12 ± 3.2 E-12	1.3 E-11 ± 1.1 E-11
	Li^+/K^+ = 1/50	2.1 E-11 ± 2.3 E-12	5.6 E-12 ± 4.0 E-12	1.5 E-11 ± 1.2 E-11
	Li^+/K^+ = 1/10	2.3 E-11 ± 2.1 E-12	7.3 E-12 ± 5.2 E-12	1.8 E-11 ± 1.7 E-11
	Li^+/K^+ = 1/2	2.4 E-11 ± 1.7 E-12	1.0 E-11 ± 7.5 E-12	1.8 E-11 ± 1.0 E-11
	Li^+	4.2 E-11 ± 6.0 E-12	1.1 E-11 ± 5.2 E-12	2.6 E-11 ± 1.9 E-11
D ratios *vs.* K^+ D_x / D_K	K^+	1	1	1
	Li^+/K^+ = 1/50	1.2 ± 0.18	1.8 ± 1.0	1.5 ± 0.83
	Li^+/K^+ = 1/10	1.3 ± 0.23	2.4 ± 1.6	1.9 ± 1.4
	Li^+/K^+ = 1/2	1.4 ± 0.29	3.2 ± 2.1	2.4 ± 1.9
	Li^+	2.3 ± 0.38	3.5 ± 1.9	3.0 ± 1.6
**Deintercalation**				
	Conditions	Dilute[a]	Concentrated[a]	Total[b]
Average diffusion coefficient D (cm^2/s)	K^+	1.9 E-11 ± 1.3 E-11	7.0 E-12 ± 6.9 E-12	1.3 E-11 ± 1.1 E-11
	Li^+/K^+ = 1/50	4.5 E-11 ± 1.8 E-11	1.4 E-11 ± 9.7 E-12	2.9 E-11 ± 2.9 E-11
	Li^+/K^+ = 1/10	4.3 E-11 ± 1.5 E-11	2.0 E-11 ± 1.6 E-11	3.1 E-11 ± 3.0 E-11
	Li^+/K^+ = 1/2	3.4 E-11 ± 2.0 E-11	2.1 E-11 ± 1.6 E-11	2.7 E-11 ± 2.1 E-11
	Li^+	5.4 E-11 ± 1.3 E-11	2.7 E-11 ± 2.1 E-11	4.0 E-11 ± 3.3 E-11
D ratios *vs.* K^+ D_x / D_K	K^+	1	1	1
	Li^+/K^+ = 1/50	2.3 ± 1.1	1.8 ± 0.44	2.1 ± 0.96
	Li^+/K^+ = 1/10	2.4 ± 1.0	2.5 ± 0.64	2.4 ± 1.0
	Li^+/K^+ = 1/2	2.4 ± 1.2	2.8 ± 0.67	2.4 ± 1.2
	Li^+	3.4 ± 1.9	3.7 ± 0.93	3.4 ± 1.9

[a] Definition of dilute and concentrated region can be found in Figure 5c.

[b] Total average of diffusion coefficients at all potentials, both dilute and concentrate regions are included.

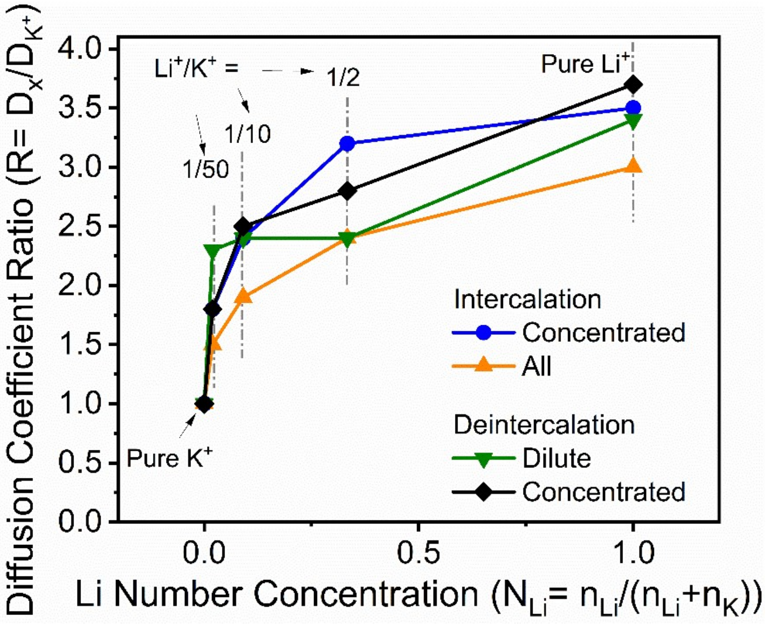

Figure S7. The ratio of diffusion coefficient at various Li^+ concentrations *vs.* that of the pure K^+ system. Diffusion coefficient ratio $R = D_x/D_K$; Li number concentration $N_{Li} = n_{Li} / (n_{Li} + n_K)$. All experiments were tested in 0.1 M $LiBF_4$, KPF_6 or $LiPF_6$ in PC-EC, on 4.9 mm^2 FLG working electrode at 1 mVs^{-1}.

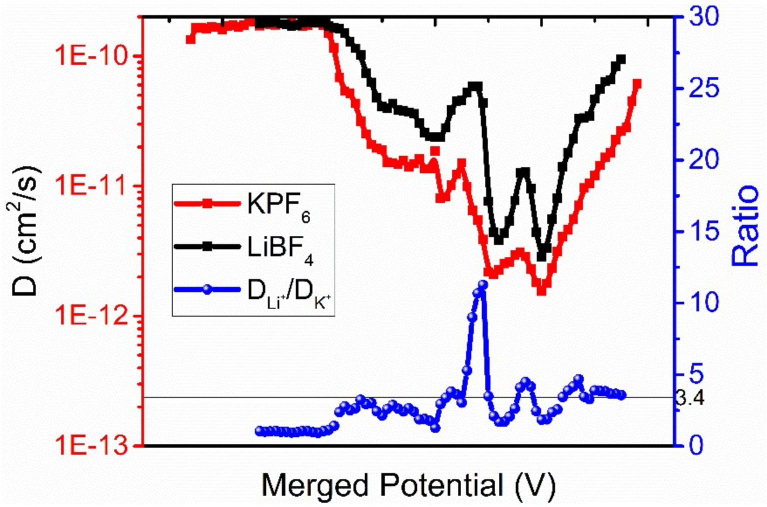

Figure S8. Example calculation method of diffusion coefficient ratio between different co-intercalation system and pristine K^+ at various stages (merged potentials). The example used here is $D_{Li}{}^+$ / $D_K{}^+$, the inserted lines represent the average value of 3.4.
